# Prediction of SCA Scores in Specialty Coffee Using Machine Learning

**DOI:** 10.1111/1750-3841.70946

**Published:** 2026-02-28

**Authors:** Gabriel Rezende Ferraz, Felipe André Oliveira Freitas, Harim H. Baldi, Natally Ferreira Lima, Gabriela Maria Rodrigues

**Affiliations:** ^1^ Universidade de Sao Paulo (USP) Piracicaba Brasil

## Abstract

Coffee is a major global commodity, with specialty coffees valued for their quality, assessed through standardized sensory protocols. The SCA (Specialty Coffee Association) score is a key indicator of commercial value, but sensory evaluation is resource‐intensive and subject to variability. This study developed predictive models to estimate SCA scores from processing and production‐related variables collected between 2019 and 2023, covering reception, fermentation, pulping, washing, drying, storage, and contextual production information. Random Forest (RF) and XGBoost (XGB) regression algorithms were applied using three approaches: complete variable set, Principal Component Analysis (PCA), and selection of the seven most relevant variables. The RF model with all variables achieved the best performance (MAE = 0.80; RMSE = 1.03; R^2^ = 0.53). However, models using only seven predictors achieved nearly equivalent results (MAE = 0.81; RMSE = 1.06; R^2^ = 0.50), with RF and XGB showing RMSE around 1.05 and R^2^ above 0.50. PCA‐based models performed worse. In conclusion, variable selection proved more efficient and robust than PCA, enabling moderate but practically relevant prediction of SCA scores with reduced model complexity in specialty coffee production.

**Practical Applications:**

This research shows that machine learning models can help predict coffee quality scores using processing data. Such tools may support producers and cooperatives in monitoring quality earlier and more efficiently, reducing reliance on extensive sensory tests and improving decision‐making in specialty coffee production.

## 1 Introduction

Coffee represents one of the main agricultural products in Latin America, standing out especially in Brazil and Colombia, global leaders in the production and export of the bean (ICO 2024). Recently, the global coffee consumer market has grown, driven not only by traditionally consuming regions such as Europe and the United States, but also by emerging markets such as China, which has gradually replaced part of its tea consumption with coffee, reflecting cultural proximity to Western habits (Monte et al. 2017).

In this scenario of increasing demand, the specialty coffee segment stands out, recognized for its high added value resulting from its distinctive sensory characteristics. However, the qualitative evaluation of these coffees, traditionally based on the sensory scoring method developed by the Specialty Coffee Association (SCA), presents significant challenges, such as the subjectivity inherent to sensory analysis and the inter‐ and intra‐evaluator variability. These limitations may compromise the efficiency and reliability of the evaluation process and, consequently, affect the transparency of commercial negotiations, fair remuneration to producers, and the quality perceived by final consumers (Thomas et al. 2017).

It is therefore justified to adopt analytical approaches capable of objectively anticipating the coffee score based on processing characteristics, prior to formal sensory evaluation. Statistical techniques and machine learning models such as Random Forest and XGBoost make it possible to predict the SCA score and identify the processing aspects that most influence final quality (Liakos et al. 2018). The database used in this study is composed of Colombian coffee samples, with variables related to post‐harvest processing and sensory evaluation, collected between 2019 and 2023.

Although previous studies have applied machine learning primarily to *classification* tasks—such as distinguishing between specialty and traditional coffees or categorizing sensory acceptance levels based on physicochemical and environmental characteristics (Adiwijaya and Sarno 2024; Santos et al. 2020; Fatan et al. 2025; Aurum et al. 2023)—most of this literature treats coffee quality as a discrete outcome. In contrast, investigations that model the SCA score as a *continuous* response variable, aiming to predict its exact value from objective processing and post‐harvest variables, remain scarce. This gap is particularly evident in the Latin American context, where few studies have explored regression‐based machine learning approaches to capture the fine‐grained variability inherent in SCA sensory evaluations.

This study proposes robust predictive models (Random Forest and XGBoost) combined with Principal Component Analysis (PCA) to estimate the SCA score of Colombian coffees, comparing two dimensionality reduction strategies: transformation of the original variables through PCA and selection of the most relevant variables by the algorithms. The aim is to develop a predictive model that increases market transparency, supports commercial decisions, and promotes fair remuneration for producers.

Thus, the motivation of this study lies in the need to improve the objectivity of qualitative evaluations of specialty coffees, providing predictive tools that anticipate sensory quality and support more assertive and transparent commercial decisions, contributing to the competitiveness and economic sustainability of the coffee sector.

Therefore, this work aimed to develop predictive models to estimate the SCA score in Colombian coffee lots, using Random Forest and XGBoost combined with PCA and variable importance selection, offering an efficient tool for the coffee sector, reducing subjectivity in evaluations, and supporting commercial decision‐making.

## 2 Materials and Methods

### 2.1 SCA Method for Special Coffee Qualification

Coffee quality was measured using a method developed by the Specialty Coffee Association (SCA), called the SCA Protocol, which can be applied by any company or people that obtain the certificate provided by the association. It consisted of a scale from zero to one hundred points determined by ten attributes rated from zero to ten, as shown in Table 1, which presents only scores given to coffees that belong to the special class.

**TABLE 1:** Special coffee classification according SCA protocol.

6.00 – Good	7.00 – very good	8.00 – excellent	9.00 – outstanding
6.25	7.25	8.25	9.25
6.50	7.50	8.50	9.50
6.75	7.75	8.75	9.75

*Note*: This table is adapted from the Specialty Coffee Association of America—Cupping Protocols (2005).

For a coffee batch to be classified as specialty, it had to meet three evaluation criteria, divided into two types of physical analysis and one sensory analysis. The physical tests involved identifying problems in the raw coffee sample and the presence of problematic beans (coffee beans with visible defects, often resulting from problems in the growth season, harvest, or processing) in the roasted coffee sample. A sensory test was performed by tasting the coffee, with the evaluation criterion being the score obtained according to specific quality protocols (National Rural Learning Service—SENAR 2017).

Although there are other protocols to assess coffee quality, the SCA protocol is the globally adopted standard. The attributes that comprise the protocol are:

1. Fragrance and aroma: the first refers to the smell of the freshly ground dry powder and the second to the already moistened drink;
2. Flavor: refers to the taste of the drink and the classification attributed to this characteristic;
3. Aftertaste: refers to the sensation that remains after consuming the liquid;
4. Acidity: refers to the type and intensity of acidity;
5. Body: refers to the quantity of essential oils in the drink;
6. Uniformity: is the measure adopted when analyzing five different cups of the same coffee;
7. Absence of defects: refers to the evaluation of strange flavors in the drink;
8. Minimum sweetness: this characteristic varies according to the coffee roast;
9. Balance: refers to the combination of flavor, acidity, and body;
10. Final concept: refers to the taster's impression of the coffee (SCA 2025).

If any of the attributes receives a score lower than six, the coffee is no longer considered specialty. According to interviews with Colombian negotiators, coffees with a score higher than 8.5 are generally destined for export at a higher value (SCA 2024).

It is important to highlight that coffee traders generally purchase lots without prior knowledge of the SCA score, which is only assigned at the export stage by independent professionals responsible for sensory evaluation and hired by the intermediary company between the grower and the international buyer. However, during the post‐harvest stages, it is possible to identify attributes that correlate with the final score of the lot. Thus, the SCA score reflects the sensory quality of the product as a result of a set of agronomic and post‐harvest practices. Therefore, the producer's main strategy for improving the quality and score of their coffees is to improve these processes.

### 2.2 Dataset

To develop this research, a database containing observations of coffee lots and their respective SCA scores was used. The collected attributes are described in Table 2.

**TABLE 2:** Collected attributes by coffee batch.

Variable (description)	Variable (description)
**Phase: Receipt** Brix—sucrose content in fruits	**Correction and washing** Beginning—wash start
pH—acidity level	End—wash end
Temperature—fruit temperature	Brix—sucrose content
Kind—coffee type	pH—acidity level
Rejection by floats (%)	Temperature—process temperature
Ripe fruits (%)	Brooked—broken grains (%)
Unripe fruits (%)	Borer—borer‐damaged grains (%)
Fruit defects—global fruit defects	**Phase: Drying (natural)** Beginning—drying start
Grain defects—specific grain defects	End—drying end
Overripe—overripe grains (%)	Final moisture—moisture content
Appearance—general appearance	Location—natural drying site
Dough temperature—coffee mass temperature	**Phase: Drying (mechanical)** Location—mechanical drying equipment/location
**Phase: Cherry fermentation** Beginning—fermentation start time	Beginning—drying start
End—fermentation end time	End—drying end
Kind—fermentation type	Final moisture—moisture content
**Phase: Depulp** Beginning—depulping start	**Phase: Storage** Bag—number of bags
End—depulping end	Weight—sample weight (kg)
Brix—sucrose content	Delivery date—delivery date
pH—acidity level	Location—storage location
Temperature—process temperature	Moisture—moisture content
Brooked—broken grains (%)	GrainPro—GrainPro bags used
Borer—borer‐damaged grains (%)	Jute—jute bags used
**Phase: Fermentation (depulped)** Beginning—mucilage fermentation start	Marking—identification
End—mucilage fermentation end	Dry coffee rejection—rejected dry coffee
Kind—fermentation type	Coffee Status—quality check
Substrate type	
Actions	
	

STUDY DATA. Attributes collected by coffee batch. 2025.

STUDY DATA. Attributes collected by coffee batch. 2025.

Table 2 shows that these batches were monitored through the harvested coffee processing stages, including reception, cherry fermentation, depulping, pulp fermentation, correction and washing, natural drying, mechanical drying, and storage. During this process the pH, temperature, sugar content, moisture, and the time the batch spent in each stage, among other attributes described in Table 2, were measured. The batch differed in coffee origin and variety; however, this study focused on variables collected during processing.

**FIGURE 1:**
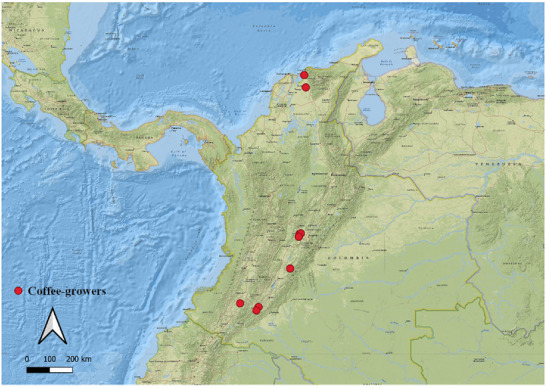
Geographical arrangement of samples used in the study. Source: From the authors, 2025.

The samples were collected from different coffee‐growing regions in Colombia, as illustrated in Figure 1. The red dots indicate the coffee beans' origins, distributed throughout the Andes Mountains and in transition zones to the coastal plain, encompassing different altitudes, microclimates, and soil types. This geographic dispersion ensures the representation of diverse physicochemical and sensory profiles, which are essential for assessing the effect of environmental conditions on final quality according to the SCA protocol.

### 2.3 Data Processing

The dataset had 59 predictor variables to be correlated with the SCA score initially, containing information collected from each batch in its reception, fermentation, depulping, fermentation, washing, drying, and storage stages. The 22 variables used in the modeling are described in Appendix A, with their respective database names and summarized definitions. The variables considered were: brix_entrada, ph_entrada, temperatura, cereja_sana, n_bultos, peso_amostra_kg, frutos_maduros, umidade, temperatura_massa, umidade_final_natural, rejeicao_flutuacao, umidade_final_mecanica, rejeicao_cafe_seco, fator_rendimento, peso_liquido_kg, sca, seno_data, cosseno_data, mes_ano_sca, delta_secagem_natural, delta_fermentacao_cc, and variedade, a total of 2.192 observations. The fields consist of measurements of pH, sugar concentration, sample temperature, weight, moisture, and minutes spent in each processing stage. Subsequently, new information was generated with the differences in pH, sugar concentration, and temperature between the different stages. The observation dates range from July 2019 to January 2023. Additionally, the batch receipt date column was transformed into two cyclical columns to capture seasonal patterns, using the equations:

(1) $$sen=sen\left(2\times \pi \times t_{norm}\left(t\right)\right)$$

(2) $$cos=cos\left(2\times \pi \times t_{norm}\left(t\right)\right)$$

where t is the numerical value of the batch receipt date (day of the year) and $t_{norm}\left(t\right)$ = t / T, where T is the maximum value of t in the data set (complete period considered).

The categorical variable “variedade” (variety) was converted into a numerical feature using a supervised CatBoost‐style target encoding strategy, designed to preserve ordinal information while avoiding high‐dimensional representations such as one‐hot encoding. To mitigate information leakage, the encoding was computed in an ordered manner prior to model training: the dataset was randomly shuffled, and for each observation, the encoded value corresponded to the mean SCA score calculated from previous observations of the same variety in the shuffled sequence. When a given variety appeared for the first time and no prior observations were available, the global mean SCA score was used as a fallback value.

This ordered target encoding allows the model to capture the historical association between coffee variety and sensory quality while ensuring that the encoded value for each observation does not incorporate its own response. Although the encoding was not recomputed within each cross‐validation fold, this procedure reduces dimensionality and limits direct target leakage, providing a compact and informative representation of the “variedade” variable, which showed strong influence across all modeling scenarios.

Subsequently, a data consistency analysis was performed, removing variables with more than 50% missing data. In addition, outliers were excluded from the variables using the Interquartile Range (IQR) method, which uses the lower (Q1) and upper (Q3) quartiles of the distribution to define limits for extreme values. According to Tukey (1977), values ​​are considered outliers when they fall outside the range defined by:

(3) $$x=outlier,\;if\;x_{i}\;\langle \;Q1\;-\;1,5\times IQ\;or\;x_{i}\;\rangle \;Q3+1,5\times IQR$$

where $x_{i}$ the i‐th observed value, and IQR=Q3−Q1 the interquartile range. The IQR criterion was deliberately chosen because it is non‐parametric and robust to skewed distributions, which are common in agricultural and sensory datasets. Importantly, this method reduces the influence of extreme observations likely associated with measurement errors or atypical processing conditions, while preserving the central structure of the data and avoiding overly aggressive trimming of genuinely informative variability. As such, it provides a balanced approach for improving model stability without systematically discarding meaningful extreme values. Finally, missing values ​​in numerical variables were filled with the median of each variable, since the algorithms used do not accept missing data in their modeling structure.

### 2.4 Structuring Predictive Modeling Scenarios

In this study, three distinct approaches were considered for using the dataset in training the *Random Forest* (RF) (Breiman 2001) and *XGBoost* (XGB) models (Chen et al. 2016).

Scenario 1 – Model with All Variables: the complete set of remaining variables was used after consistency analysis and exclusion of outliers (RF_ALL, XGB_ALL).

Scenario 2 – Dimensionality Reduction via PCA: Principal Component Analysis (PCA) was applied to the predictor variables, with prior data standardization. The number of retained principal components was determined based on the Kaiser criterion, and the selected components were used as input to the models (RF_PCA, XGB_PCA).

Scenario 3 – Variable Selection by Importance: a reduced predictor base composed only of the most relevant variables was used, which were obtained from training in Scenario 1, considering the values ​​assigned by each algorithm to the most influential attributes, both in RF and XGB (RF_IMP, XGB_IMP).

#### 2.4.1 Principal Component Analysis

Principal Component Analysis (PCA) was used for dimensionality reduction to identify the attributes that contribute most to data variance and thus generate a new set of variables (Hongyu, Sandanielo, and Junior 2016). The predictor variables were first standardized, after which the covariance matrix was computed and its corresponding eigenvalues and eigenvectors were obtained. Principal components were then selected according to the Kaiser criterion (Kaiser 1960), which retains only components with eigenvalues greater than 1, indicating that the component explains more variance than an individual standardized original variable. This criterion was chosen for its simplicity, interpretability, and widespread use in exploratory analyses, particularly when the goal is to achieve dimensionality reduction while preserving the dominant variance structure of the data. Finally, the data were projected onto the selected principal component space and used as inputs for the predictive models.

#### 2.4.2 Random Forest

The Random Forest (RF) regression model (Breiman 2001) was applied in this study to predict the SCA score of coffees, using attributes obtained during the processing steps as predictor variables. The algorithm was implemented in the R program, using the train function of the caret package. To adjust the model, k‐fold cross‐validation (Burman 1989) was used, with k = 5, to optimize the hyperparameters using the smallest root mean square error (RMSE) as the metric during training. The optimal Random Forest configuration was obtained with *mtry* = 5 and *ntree* = 300.

After training the full model, an analysis of the importance of the predictor variables was performed. This analysis allowed us to identify which variables had the greatest influence on the SCA score, supporting the interpretation of the results and highlighting which processing attributes were most relevant to the final coffee quality.

#### 2.4.3 The Extreme Gradient Boosting (XGBoost)

The Extreme Gradient Boosting (XGBoost) model (Chen et al. 2016) was implemented for regression to predict the SCA score, also using the variables obtained during the processing steps. The implementation was done in R, using the caret package. The same approach described above was adopted for hyperparameter optimization, with validation across seven partitions and the lowest RMSE as the deciding factor. The optimal XGBoost model was obtained with *nrounds* = 200, *max_depth* = 5, *eta* = 0.1, *gamma* = 0, *colsample_bytree* = 0.8, *min_child_weight* = 1, and *subsample* = 1.

Furthermore, the importance of variables was extracted based on the cumulative gain attributed to each across the trees of the final model, allowing a clear interpretation of which variables had the greatest impact on predictions.

#### 2.4.4 Model Evaluation and Comparison

Initially, the data were divided into training (80%) and testing (20%). During training, stratification was performed by discretizing the SCA variable into six ordered intervals. This stratifying variable was used to guide the formation of subsets during the cross‐validation process described above. In both models and for all scenarios, a stratified k‐fold cross‐validation strategy was adopted during training, with k = 5, to ensure greater robustness in the estimation of predictive performance and avoid information leakage. This procedure was controlled using the caret package in the R program.

The performance of the RF and XGB models was evaluated using the RMSE, mean absolute error (MAE), and coefficient of determination (R^2^) metrics, calculated based on test performance. These metrics allowed for an objective comparison between the models, reflecting their ability to predict the SCA score.

## 3 Results and Discussion

The selection of these variables was carried out based on the importance levels obtained in the initial analysis stage (Scenario 1), from training the model with the complete set of variables. In this way, it was possible to compare the performance of the models trained with all variables, with those trained using predictors selected by importance, and with principal components, thus comparing the dimensionality reduction strategies adopted in this study.

### 3.1 Scenario 1 – Model With All Variables

**FIGURE 2:**
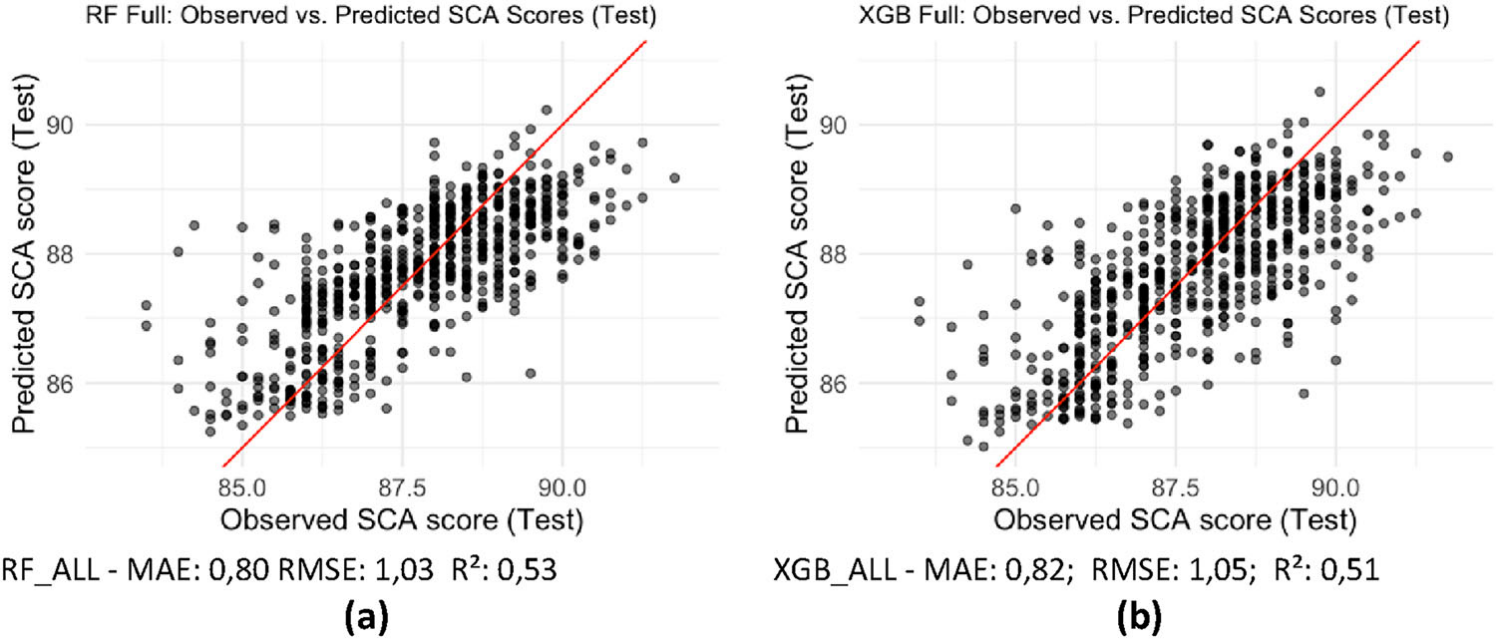
Observed versus Predicted Comparison: Random Forest and XGBoost (all variables). Source: From the authors, 2025.

In Figure 2, it is possible to observe the result of the model that used the complete set of variables remaining after the consistency analysis and the removal of outliers. Figure 2a presents the results for the RF_ALL model, which showed a mean absolute error (MAE) of 0.80 and a root mean square error (RMSE) of 1.03, indicating moderate predictive performance, as the predicted values were reasonably close to the observed ones. The coefficient of determination R^2^ of 0.53 shows that about 53% of the variation in the SCA scores was explained by the model variables. The remaining unexplained variability may be partially attributed to the inherent subjectivity of sensory evaluation. It is also observed that, in the comparison between predicted and observed values, the scatter plot reasonably followed the identity line, reinforcing the moderate nature of the predictive accuracy. But a slope was noticed at the extremes, indicating that the model tends to underestimate very high values and overestimate very low ones.

In Figure 2b, which shows the performance of XGB_ALL for the same dataset, an MAE of 0.82 and an RMSE of 1.05 were observed, with an R^2^ of 0.51. This means that, on average, the predictions of XGB_ALL deviate by about 0.82 points from the actual score, a value slightly higher than the MAE of 0.80 from RF_ALL, and that 51% of the variability of the evaluations was explained by the model, compared to 53% for RF_ALL. Visually, the scatter plot remained slanted relative to the identity line, with slightly greater dispersion compared to RF_ALL, also showing a tendency to underestimate higher values and overestimate lower ones, as observed in RF_ALL. However, although the RF_ALL and XGB_ALL models presented very close performance in terms of metrics, RF_ALL maintained a slight quantitative and visual advantage regarding the dispersion of the data.

**FIGURE 3:**
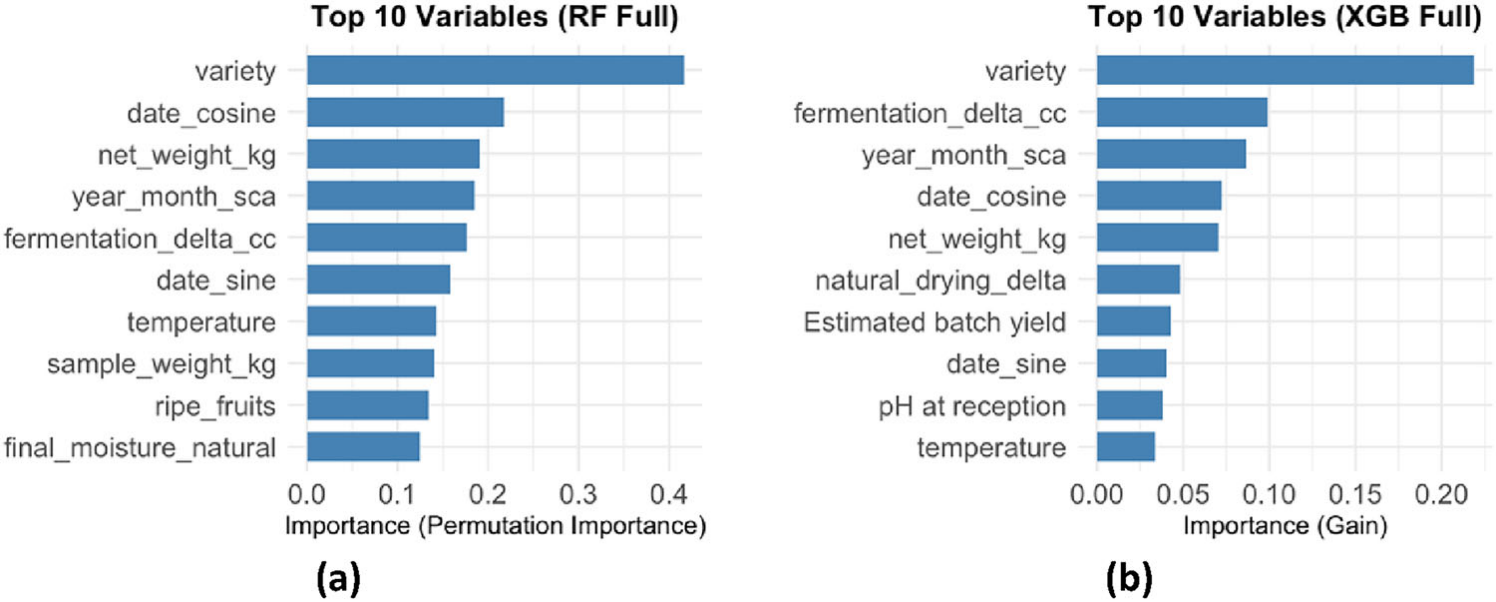
Comparative importance of predictor variables in Random Forest and XGBoost models: Scenario 1 – Model with All Variables. Source: From the authors, 2025.

Subsequently, a comparative analysis of variable importance was carried out (Figure 3), revealing that in both algorithms, “variety” was the most influential predictor of the SCA score. In the RF_ALL and XGB_ALL models, the three main variables (variety, fermentation delta (delta_fermentacao_cc), and annual mass evaluated by SCA (mes_ano_sca)) concentrated most of the explanatory power, indicating that both cultivar characteristics and fermentation conditions, as well as the soluble solids profile (Brix), were decisive for sensory quality. Net weight (peso_liquido_kg) and natural drying delta also ranked among the five most relevant in both methods, reinforcing the importance of post‐harvest processes. Finally, parameters such as the proportion of ripe fruits, sample mass, and drying temperature showed moderate influence, while the yield factor occupied lower positions.

### 3.2 Scenario 2: Dimensionality Reduction Through Principal Component Analysis (PCA)

**FIGURE 4:**
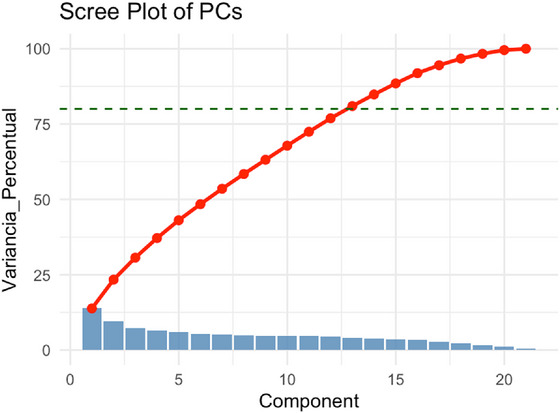
Scree Plot of the Principal Components with explained and cumulative variance. Source: From the authors, 2025.

Figure 4 shows the scree plot of the principal components, in which the blue bars indicate the variance explained individually by each principal component, and the red line represents the cumulative variance. In this study, the first nine principal components (PCs) were selected according to Kaiser's criterion, which together explained 60.53% of the total variance in the data. This approach was employed to reduce the dimensionality of the dataset while maintaining a representative proportion of the original variability for predictive purposes.

**FIGURE 5:**
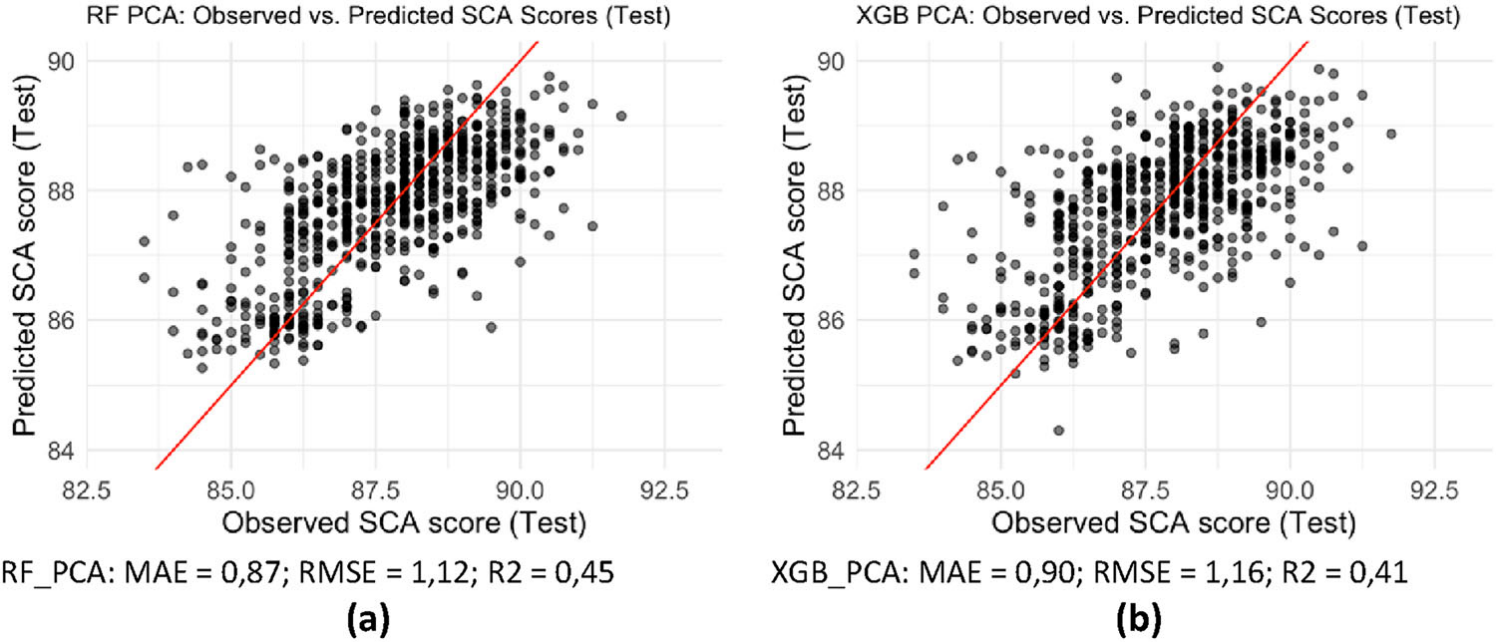
Observed versus Predicted Comparison: Random Forest and XGBoost with PCA (10 components). Source: From the authors, 2025.

In Figure 5, the results are shown for the approach that applied PCA to the predictor variables, using the first nine principal components as input to the models according to Kaiser's criterion. For the RF_PCA model (Figure 5a), the MAE was 0.87, the RMSE was 1.12, and the R^2^ was 0.45, indicating that by using only the first nine principal components, the model explained 45% of the variability in SCA scores and showed a higher root mean square error compared to the RF model that used the complete set of variables (RMSE = 1.03). In the graph, the dispersion of points around the identity line at the extremes indicates a tendency of the model to underestimate higher‐quality evaluations and overestimate lower‐quality ones.

Figure 5b presents the performance of the XGB_PCA model after applying PCA, considering the first nine components. The results indicate an MAE of 0.90, an increase in RMSE to 1.16, and a reduction in R^2^ to 0.41. This means that the model explained only 41% of the variation in SCA scores and committed larger mean squared errors compared to XGB_ALL, which used the full set of variables (RMSE = 1.05; R^2^ = 0.51), and to RF_PCA (RMSE = 1.12; R^2^ = 0.45). A greater dispersion of points around the identity line was also observed, and the behavior at the extremes suggests a tendency to underestimate higher SCA values and overestimate lower ones, similar to RF_PCA. Despite the lower metrics, the RF_PCA and XGB_PCA models displayed a visual behavior similar to the others.

**FIGURE 6:**
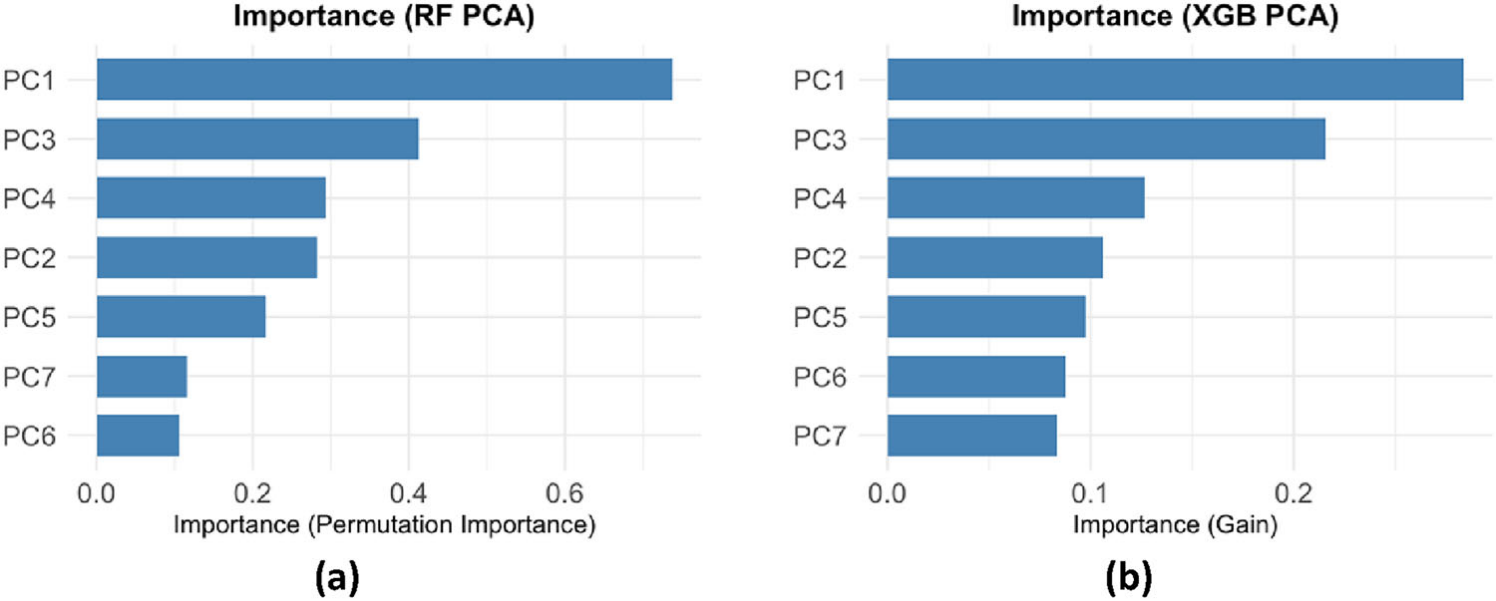
Comparative importance of predictor variables in Random Forest and XGBoost models: PCA approach. Source: From the authors, 2025.

The analysis of principal component importance (Figure 6) indicated that, for both algorithms, the first component (PC1) accounts for the largest share of predictive capacity, surpassing the second‐ranked component (PC4). Next, PC3 and PC2 concentrate the second‐ and third‐highest importance values, indicating that the linear combinations of the original variables captured in these components remain relevant for explaining the variation in SCA scores. Lower‐order components (PC8, PC6, PC7, and PC5) show decreasing and relatively close importance values, suggesting that they contribute little and are nearly equivalent. It is worth noting that there is no guarantee that the components with higher variance necessarily contain the most predictive information for the attribute of interest in either case.

### 3.3 Scenario 3: Model With Variables Selected by Importance (Feature Selection)

The third approach consisted of the exclusive use of the most relevant variables identified in the complete RF_ALL and XGB_ALL models, aiming to assess predictive performance in scenarios with lower dimensionality. In both approaches, seven variables stood out as the most influential for predicting coffee quality (SCA score): variety, mes_ano_sca, delta_fermentacao_cc, peso_liquido_kg, cosseno_data, delta_secagem_natural, and frutos_maduros (in XGB_ALL) or seno_data and peso_amostra_kg (in RF_ALL). The variable “variety” was consistently the most relevant, reflecting the strong genetic effect on the composition and final quality of the batch. Variables related to processing time (delta_fermentacao_cc, delta_secagem_natural) and harvest period (mes_ano_sca, seno/cosseno_data) also showed importance, highlighting the influence of the agricultural calendar and post‐harvest practices on sensory quality. In addition, variables associated with mass and batch yield (peso_liquido_kg, peso_amostra_kg) reinforce the relationship between physical attributes and the final score assigned to the coffee.

**FIGURE 7:**
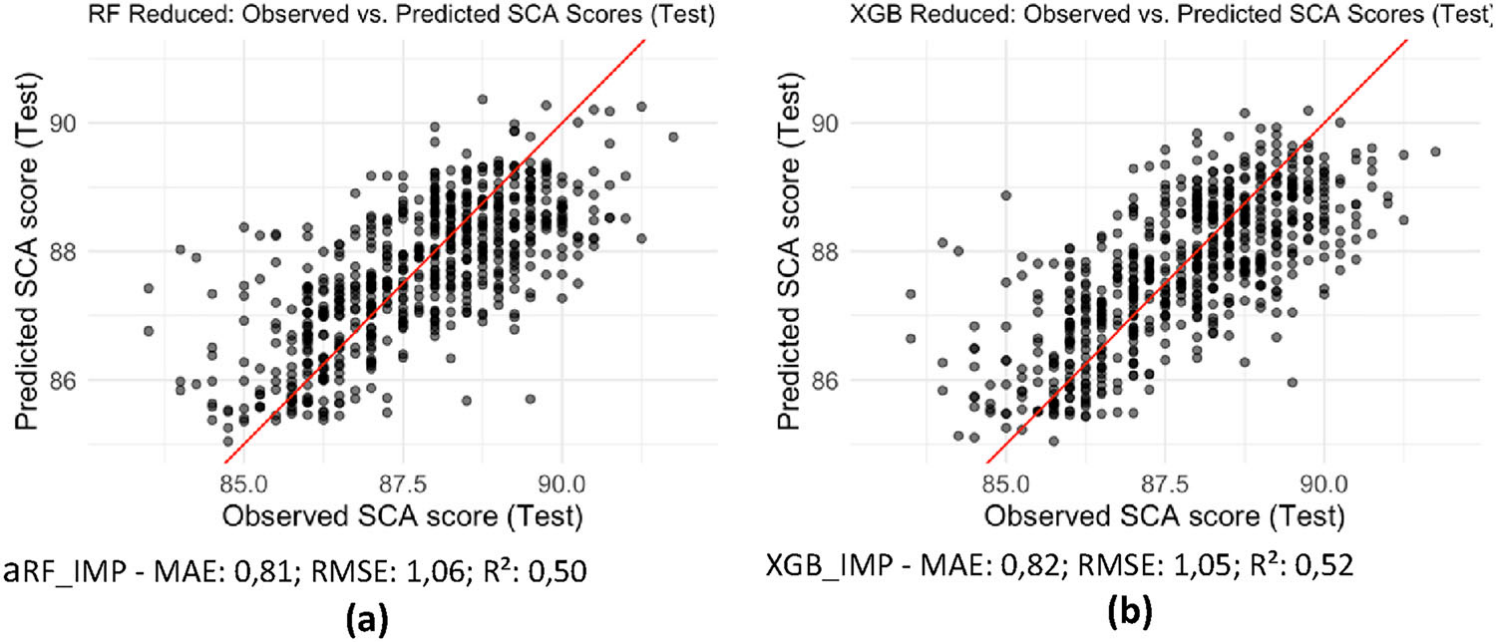
Observed versus Predicted Comparison: Random Forest and XGBoost with the 7 most important variables. Source: From the authors, 2025.

Using the set of the seven most important variables, the reduced models were estimated, as shown in Figure 7. The RF_IMP model (Figure 7a) presented an MAE of 0.81, an RMSE of 1.06, and the R^2^ of 0.50, values slightly lower than those obtained with the full set of variables (RF_ALL and XGB_ALL). In the observed versus predicted plot, most points are concentrated along the identity line, demonstrating adequate fitting capacity given the reduced set of predictors. Nevertheless, underestimation of the highest scores and overestimation of the lowest scores are still observed at the extremes of the scatter plot, as in the previous scenarios. These results indicate that, even with only seven variables, the model maintains its performance, simplifying the structure without substantially degrading predictive performance.

In Figure 7b, which shows the XGB_IMP model (considering the set of the seven most important variables in XGB_ALL), the MAE was 0.82, the RMSE was 1.05, and the R^2^ was 0.52, performance very similar to that obtained with the full set in XGB_ALL (MAE = 0.82; RMSE = 1.05; R^2^ = 0.51). Visually, the dispersion around the identity line is more pronounced, and at the extremes, XGB_IMP also tends to underestimate higher scores and overestimate lower ones when restricted to the seven variables. However, these results suggest that, as with RF, the exclusion of predictors did not compromise the fitting capacity and performance of the XGB model.

**FIGURE 8:**
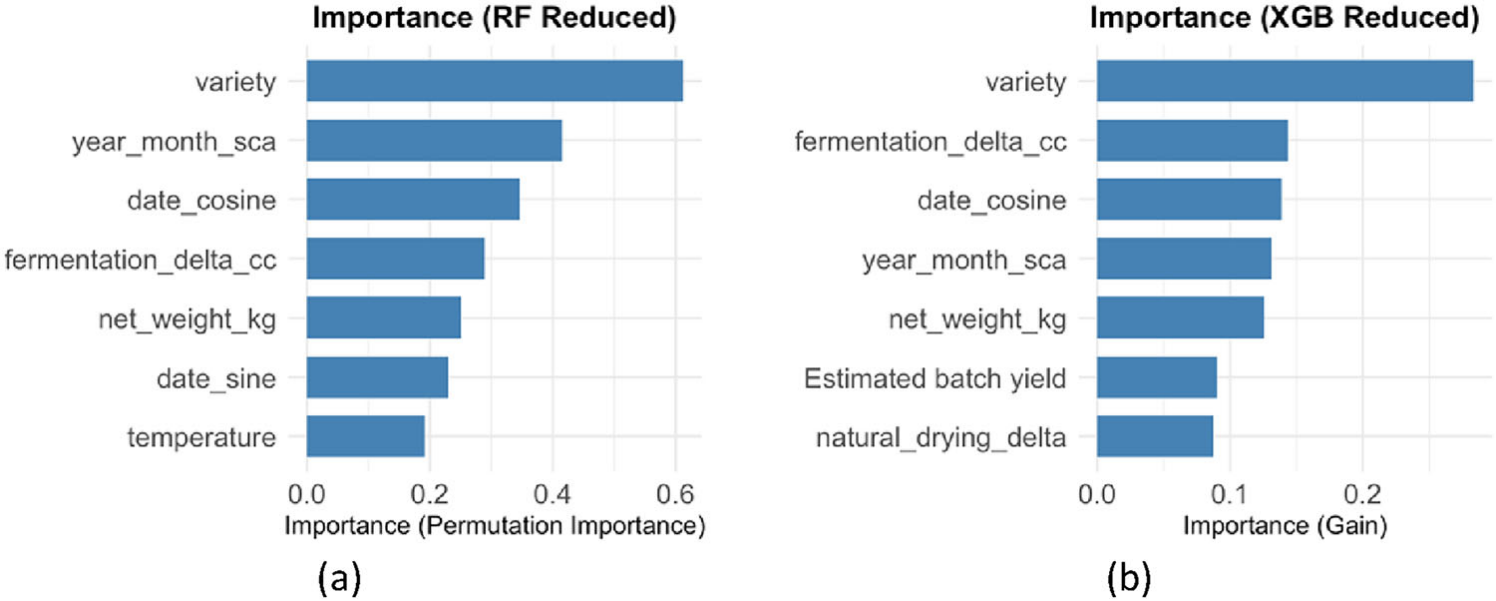
Comparative importance of predictor variables in Random Forest and XGBoost models: Reduced approach. Source: From the authors, 2025.

In this approach (Figure 8), the variable “variety” remains the dominant predictor in both algorithms, followed by “mes_ano_sca” and “delta_fermentacao_cc.” In RF_IMP, the importance ranking decreases in a pattern similar to XGB_IMP, with “peso_liquido_kg,” “cosseno_delta,” and “delta_secagem_natural” also exerting influence, while “peso_amostra_kg” appears as the least relevant. In XGB_IMP, a greater concentration of weight is observed in the first predictor (variety), with a sharper drop for the second‐ranked variable, “peso_liquido_kg”.

## 4 Discussion

This study evaluated the comparative performance of two machine learning algorithms, Random Forest (RF) and XGBoost (XGB), for predicting coffee quality according to the SCA protocol. Three analytical approaches were adopted: (i) use of the complete set of variables after data cleaning and outlier removal, (ii) dimensionality reduction through Principal Component Analysis (PCA), using the first ten components, and (iii) selection of the seven most relevant variables identified through importance analysis.

Among the evaluated approaches, the RF model trained with the complete set of variables achieved the highest overall performance, followed closely by XGB. Given the inherent subjectivity and inter‐evaluator variability of sensory analysis under the SCA protocol, the moderate R^2^ values observed (≈ 0.50–0.53) indicate that approximately half of the variability in SCA scores can be explained by the available predictors, which is consistent with the complexity of the response variable. These results highlight the capacity of ensemble algorithms to capture nonlinear relationships and complex interactions among agronomic and processing variables, even under realistic limits of predictability (Breiman 2001; Chen and Guestrin 2016). Previous studies have shown that physicochemical and processing attributes, even with marginal correlation, can emerge as important through interactions captured by decision trees (Kim 2022).

When applying PCA, a reduction in the explanatory capacity of the models was observed, with an R^2^ of 0.45 in RF_PCA and 0.41 in XGB_PCA, along with an increase in RMSE. As PCA is an unsupervised method, it prioritizes variance preservation rather than predictive relevance with respect to the response variable, which may exclude low‐variance components that are nonetheless associated with sensory quality (Guyon and Elisseeff 2003). Accordingly, while PCA remains useful for exploratory analyses and collinearity assessment, it proved less effective as a dimensionality reduction strategy for maximizing predictive performance in this context.

On the other hand, the direct selection of the seven most important variables in Scenario 3 yielded promising results. Both RF and XGB models trained on the reduced predictor set retained error levels and explanatory power comparable to those obtained with the full variable set, demonstrating that a more parsimonious approach can maintain predictive consistency while improving interpretability and computational efficiency (Guyon and Elisseeff 2003). Variables related to cultivar, processing conditions, and post‐harvest handling played a relevant role in explaining sensory scores, supporting the efficiency of the simplified models (Kim 2022).

Another relevant point concerns the practical applicability of the models, especially in agricultural, industrial, and commercial contexts. The proposed models should be interpreted as complementary decision‐support tools rather than substitutes for formal sensory analysis, providing early and approximate estimates of SCA scores that may support post‐harvest and commercialization decisions (Kim 2022; Putra et al. 2023).

Previous studies have reported inter‐ and intra‐panel variability in SCA scores on the order of approximately one point, depending on panel composition, calibration, and evaluation conditions. In this context, the RMSE values around 1.0 observed in this study are comparable to the intrinsic variability of sensory assessment itself, reinforcing the practical relevance—but also the limitations—of the proposed models.

Overall, this work contributes to the literature by systematically comparing dimensionality reduction strategies for predicting coffee sensory quality based on processing and production‐related information, offering a transparent and replicable framework for future studies.

Several limitations should be acknowledged. The analysis was restricted to two machine learning algorithms and to data from a single producing region and harvest context, which may limit generalizability. In addition, no direct chemical descriptors or panel‐level sensory information were included. Future research may benefit from integrating chemical profiling, nonlinear dimensionality reduction methods, hybrid ensemble strategies, and multi‐region datasets to further advance the prediction of coffee sensory quality.

## 5 Conclusion

The results of this study show that, in predicting coffee quality according to the SCA protocol, the variable selection approach significantly influences the performance of machine learning models. Dimensionality reduction via PCA, although useful for mitigating collinearity, compromised the retention of essential predictive information, resulting in the lowest performances observed. The selection of a subset of seven most relevant variables proved effective for both models (Random Forest and XGBoost), preserving or slightly improving predictive performance while producing leaner and more interpretable models. These results indicate that parsimonious models can achieve predictive consistency comparable to more complex specifications, particularly when interpretability and computational efficiency are desired. Variables related to cultivar, processing conditions, and production context consistently emerged as influential within the analyzed dataset, reinforcing their relevance for decision‐support applications in specialty coffee production.

The performance between RF and XGB was similar, with a slight advantage for RF_ALL in the complete set and XGB_IMP in the reduced subset, indicating that the choice of algorithm depends on the objective: greater overall robustness with RF or a leaner and more efficient model with XGB.

In summary, this work demonstrates that importance‐based feature selection offers an effective balance between model simplicity and predictive performance, while reinforcing that machine learning models should be viewed as complementary tools to support, rather than replace, human sensory evaluation.

## Author Contributions

**Gabriel Rezende Ferraz**: conceptualization, methodology, software, data curation, writing – review and editing, writing – original draft. **Felipe André Oliveira Freita**: conceptualization, methodology, software, writing – review and editing, writing – original draft. **Harim H. Baldi**: conceptualization, methodology, software, writing – review and editing; writing – original draft. **Natally Ferreira Lima**: conceptualization, methodology, software, writing – review and editing, writing – original draft. **Gabriela Maria Rodrigues**: conceptualization, methodology, software, writing – review and editing, writing – original draft, project administration.

## Conflicts of Interest

The authors declare no conflicts of interest.
